# Investigating Gut Permeability in Animal Models of Disease

**DOI:** 10.3389/fphys.2018.01962

**Published:** 2019-01-15

**Authors:** Marianela González-González, Camilo Díaz-Zepeda, Johana Eyzaguirre-Velásquez, Camila González-Arancibia, Javier A. Bravo, Marcela Julio-Pieper

**Affiliations:** Grupo de NeuroGastroBioquímica, Instituto de Química, Facultad de Ciencias, Pontificia Universidad Católica de Valparaíso, Valparaíso, Chile

**Keywords:** gut, permeability, barrier, epithelium, gastrointestinal disorders

## Abstract

A growing number of investigations report the association between gut permeability and intestinal or extra-intestinal disorders under the basis that translocation of gut luminal contents could affect tissue function, either directly or indirectly. Still, in many cases it is unknown whether disruption of the gut barrier is a causative agent or a consequence of these conditions. Adequate experimental models are therefore required to further understand the pathophysiology of health disorders associated to gut barrier disruption and to develop and test pharmacological treatments. Here, we review the current animal models that display enhanced intestinal permeability, and discuss (1) their suitability to address mechanistic questions, such as the association between gut barrier alterations and disease and (2) their validity to test potential treatments for pathologies that are characterized by enhanced intestinal permeability.

## Introduction

Many clinical disorders are characterized or accompanied by GI alterations, including symptoms such as discomfort or pain, bloating and altered motility which can negatively impact a patient’s quality of life. For example, irritable bowel syndrome, a chronic, relapsing GI problem (Spiller et al., 2007) with a high overall prevalence ranging from 10 to 20% of the population (Guarner et al., 2008), is also highly comorbid with other pathologies including depression and anxiety, cardiovascular disease and fibromyalgia (Cole et al., 2006; Mussell et al., 2008; Gros et al., 2009; Faresjo et al., 2013). In the past decade, this intricate connection between the GI and other systems has received deep attention allowing for the coining of terms such as gut-brain axis (Gonzalez-Arancibia et al., 2016), gut-liver axis (Federico et al., 2016), or gut-kidney axis (Khoury et al., 2016). Likewise, the number of papers addressing the contribution of intestinal microbiota (which may be considered “an organ within an organ”) aspects to the above axes has increased exponentially in the last few years.

The adequate relationship between GI physiology and overall homeostasis involves a variety of delicate functions; among these, the participation of gut motility, intestinal epithelial secretion and visceral perception mechanisms in association to systemic alterations have been somewhat explored (Duclos et al., 1991; Lackner et al., 2004; Zheyu et al., 2007; Yamamoto et al., 2008; Diebel et al., 2015; Yamamoto-Furusho et al., 2015). However, the contribution of other complex responses such as those regulated by the ENS or the intestinal epithelial barrier to the development of GI and extraintestinal disorders are still largely unknown. Regarding the association between gut barrier modifications and disease, abundant but mostly observational data is available (see Table 1). These evidences strongly suggest the involvement of an altered barrier in either the origin or the manifestations of these diseases, although the exact mechanisms are yet to be investigated. A few recent temporal, genetic and twin studies have looked into the potential role of a dysfunctional gut barrier in the onset of disease (Uhlig et al., 2014; Tornai et al., 2017; Keita et al., 2018). Still, the pathogenesis of inflammatory gut diseases is unclear, and although the previous reports somewhat point to barrier-associated factors (including genetic predisposition and unfavorable luminal environment conditions) further exploration of this hypothesis is still needed. Appropriate animal models may allow addressing such mechanistic questions, while also allowing to test potential treatments for pathologies that are characterized by enhanced intestinal permeability.

**Table 1:** Disorders associated with increased gut permeability.

**Gastrointestinal disorders associated with gut barrier alterations**	
Inflammatory bowel disorders	Soderholm et al., 1999; Vivinus-Nebot et al., 2014
Celiac disease	Heyman et al., 2012
Food allergies	Perrier and Corthesy, 2011
Infectious diarrhea	Sharpstone et al., 1999; Hoque et al., 2012
Irritable bowel syndrome	Camilleri et al., 2012
**Extraintestinal disorders and conditions associated with gut barrier alterations**	
Critical illness and multiple organ dysfunction syndrome	Zhang et al., 2010
Burn injury	Ziegler et al., 1988; Earley et al., 2015
Heart failure	Sandek et al., 2007
Renal failure	Magnusson et al., 1991; Vaziri et al., 2013
Liver disease	Schnabl, 2013
Rheumatologic disorders	Ciccia et al., 2010
Dermatologic disorders	Humbert et al., 1991; Majamaa and Isolauri, 1996
Diabetes	Bosi et al., 2006; Sapone et al., 2006
Depression	Maes et al., 2008
Schizophrenia	Severance et al., 2013


## Factors Influencing the Gut Barrier Function

The GI tract has an estimated surface area of 32 m^2^ (Helander and Fandriks, 2014). Being constantly exposed to microbes and potentially damaging or immunogenic substances (i.e., bacterial, dietary and xenobiotic components and their metabolites) it requires a selective and efficient barrier function. This is composed of several main layers: more externally, the gut microbiota; a mucus gel coat acting as a physical diffusion layer; and the epithelium, responsible for secretion, absorption, endocrine and immune functions, to name a few.

Regarding the gut microbiota, it has been well shown that the absence of luminal bacteria at key developmental stages drastically affects the maturation of the gut barrier (Wagner et al., 2008; Sommer and Backhed, 2013). Animals reared in germ-free conditions display striking alterations in the morphology as well as in several immune, biochemical and biophysical parameters of the intestinal barrier. Also these germ free animals have alterations in the ENS, such as reduced neuron excitability (McVey Neufeld et al., 2013). Likewise, reduced intestinal virome diversity and altered intestinal barrier function are associated not only with infectious and autoimmune diseases but also metabolic disorders and cardiovascular disease (Carding et al., 2017).

Most bacteria and viruses do not contact the gut epithelium directly due to the presence of mucus. The intestinal mucus gel coat is produced by a subset of epithelial cells known as goblet cells and contains mucins, highly glycosylated proteins that form a viscoelastic network. Important defense functions are exerted by the intestinal mucus: it acts as a physical diffusion barrier to pathogens and contains antimicrobial peptides and immunoglobulins, produced by mucosal Paneth cells and plasma B cells, respectively (Johansson et al., 2013).

The intestinal epithelium also restricts the passage of microbes and substances from the luminal space to the systemic compartment. Its main cell type are enterocytes which absorb nutrients, water and electrolytes and can also secrete water and electrolytes. Enterocytes connect to each other through protein structures, namely desmosomes and adherens junctions, integrins and tight junctions (Daneman and Rescigno, 2009). These protein complexes are key to modulate the paracellular transport of substances across the epithelium, which represents the predominant means of gut epithelial permeability (Suzuki, 2013). Tight junctions (TJ) allow the movement of water, small solutes and electrolytes between epithelial cells, but restrict the translocation of larger molecules. TJ function is dynamic and can be regulated by factors associated to cellular stress, gut microbes and dietary compounds (Galipeau and Verdu, 2016). The transepithelial transport of compounds with high molecular weight is achieved via the transcellular pathway, which includes the processes of carrier-dependent transport and endocytosis, important for nutrient absorption. Increased permeability via the transcellular but especially the paracellular pathway can allow for excessive translocation of microbial and diet-derived molecules, potentially contributing to inflammatory conditions (Galipeau and Verdu, 2016).

Another gut epithelial cell type are enteroendocrine cells, capable of releasing endocrine mediators. They have been called “sentinels of the intestinal environment” (Worthington et al., 2018) and are able to regulate intestinal inflammation by enhancing secretion of the anti-inflammatory mediator GLP-1 (Lebrun et al., 2017). According to the authors, gut injury-associated increase in permeability is believed to allow LPS access to stimulate a subtype of enteroendocrine cells, resulting in GLP-1 secretion. This peptide would favor mucosal integrity by attenuating local and systemic inflammation.

The gut mucosa is not only composed of epithelial cells. Immersed in the intestinal epithelium are immune cells which upon contact with luminal material are able to coordinate adequate responses either to prevent microbial invasion (Li et al., 2012) or to dampen down exaggerated immune reactions (Turner, 2009). Additionally, these mucosal immune cells exert modulatory functions over the intestinal barrier; for example, T helper cell-derived cytokines can help modify the flux of molecules across tight junctions or cation pores (Turner, 2009). If microbes surpass the intestinal epithelium, they are eliminated by macrophages from the lamina propria (Kelsall, 2008) in a process characterized by the lack of a strong proinflammatory tone (Smythies et al., 2005). In addition, upon damage macrophages migrate to the injured area and promote epithelial tissue repair (Pull et al., 2005).

In addition to immune cells, the gut wall contains neurons which are another important aspect in the modulation of intestinal barrier permeability. Mediators released by the ENS, which is organized in submucosal and myenteric plexi, can modulate not only paracellular permeability, water/electrolyte transport and nutrient absorption but also wound healing, in particular the process of epithelial proliferation/differentiation (Neunlist et al., 2013).

Intestinal barrier function is critical for gut homeostasis. A breach in this barrier can be originated by improper function of one or more of its cell types, including inadequate interaction between epithelial cells associated not only to alterations of the tight junctions but also weakened intercellular adherent connections (Viggiano et al., 2015). As such, abnormal intestinal permeability is an important component in dysfunctional gut barrier-associated disorders such as IBD and celiac disease (Groschwitz and Hogan, 2009). Altered permeability has been associated to several human diseases (see Table 1) in the GI and other systems. The traditional view that defines increased permeability as a mere consequence of disease processes is now outdated; alterations in the translocation of luminal substances are currently being considered as part of disease etiopathogenesis (Keita et al., 2018). Therefore, there is increasing need for animal models of gut permeability not only to test palliative therapies but to further investigate disease etiology and subsequently propose novel pharmacological targets. Fortunately, many functional, cellular and biochemical features of the intestinal barrier are conserved among species; for example, a high degree of similarity is observed between tight junction proteins across vertebrates (Robinson et al., 2015; Brugman, 2016).

## Methods for Testing Gut Permeability and Other Markers of Barrier Disruption

When testing for intestinal permeability, a variety of parameters can be evaluated. Moreover, the fact that permeability varies along the GI tract must be taken into account, being the small intestine more permeable than the large intestine (Mateer et al., 2016). Excellent reviews are available (Wang et al., 2015; Fukui, 2016; Galipeau and Verdu, 2016) which summarize the most commonly used permeability tests for both basic and clinical research, in order to evaluate the status of epithelial barrier integrity, the pathophysiology of leaky gut alterations or to prove the effectiveness of treatment.

Briefly, methods for testing gut permeability *in vivo* involve the administration of a tracer molecule by oral gavage or intestinal instillation. Tracers commonly used are non-digestible sugars such as lactulose or mannitol, PEG, fluorescently labeled dextrans and ^51^Cr-EDTA (see Galipeau and Verdu, 2016 for applications, advantages and limitations of these and other tracers), which can be later quantified in urine or blood. The size of a tracer can indicate the probable route of permeability: for example, 4 kDa dextrans are able to pass through the paracellular route. This is also the case for mannitol and ^51^Cr-EDTA. On the other hand, larger molecules like 40 kDa HRP are commonly associated to the transcellular pathway (Galipeau and Verdu, 2016). The output in most of these techniques is a single value of permeability, which does not allow to discern which region of the GI is being affected, and therefore must be used in combination with other methods (Galipeau and Verdu, 2016). Also, factors affecting the distribution and excretion of orally administered probes, such as gastric emptying, intestinal transit, bacterial degradation, intestinal blood flow, as well as the timing of blood or urinary collection should be taken into consideration (Bjarnason et al., 1995).

Short term culture of tissue explants for *ex vivo* permeability tests has the advantage of allowing for the evaluation of very specific regions of the GI tract and also maximizes data output in expensive or lengthy treatments. However, tissue viability is a major concern and therefore incubation times no longer than 3 h are recommended (Clarke, 2009). The use of everted gut sacs to measure the transit of fluorescent probes has the advantage of not demanding specialized equipment, but larger amounts of tissue are required (3–4 cm^2^) (Moyano-Porcile et al., 2015; Eyzaguirre-Velasquez et al., 2017). In the Ussing chamber, ion transport across the intestinal wall can be measured in very small segments of tissue (1 cm^2^) but a more expensive setup is necessary. Here, the degree of TEER is considered an inverse correlate to the extent of paracellular permeability (Clarke, 2009). Although there has been some debate around whether TEER represents trans- or paracellular permeability, its close association to the tight junction protein dynamics (Srinivasan et al., 2015) makes us agree to the latter. Since the apical and basolateral sides are kept in isolated chambers, the passage of tracers can also be evaluated. FITC-dextrans, ^51^Cr-EDTA and HRP have been used to evaluate paracellular and transcellular permeability, respectively (Galipeau and Verdu, 2016). The Ussing chamber can also be used to evaluate permeability across monolayers of cultured epithelial cells (Brown and O’Grady, 2008), such as the Caco-2 cell line, which polarize under appropriate growth conditions. In cell monolayers, TEER measurements can also be performed in an ohm-volt meter (Zhao et al., 2016), a simpler device when compared to the Ussing chamber + voltage clamp apparatus.

In order to obtain comprehensive information regarding epithelial leakiness it is recommended that *in vivo* and *ex vivo*/*in vitro* tests of permeability are used in combination with the detection of permeability-associated biomarkers (Galipeau and Verdu, 2016). For example, the presence of microbial products or the corresponding antibodies in the blood is an indirect evidence of barrier breach (Tian et al., 2009; Vancamelbeke and Vermeire, 2017). These strategies allow for testing in a less invasive, non-terminal fashion but again, do not allow discerning which region of the GI is being affected. On the other hand, evaluating (a) mucosal morphology (including villus and/or crypt length, inflammatory cell infiltration, etc.) (Li et al., 2015), (b) the presence of tracer particles in pinocytic vesicles within gut absorptive cells (Worthington and Syrotuck, 1976), together with (c) tight junction/inflammatory protein and/or mRNA expression are more invasive, usually terminal strategies that provide information regarding the site and potential mechanism of damage (Steegenga et al., 2012; Hwang et al., 2013).

## Studying Gut Barrier Alterations in Animal Models

The gut barrier is structurally and biochemically conserved across species, as mentioned before. Both in humans and rodents the intestinal barrier function is highly sensitive to stress, dietary and microbiota changes. Therefore, we must consider that most animal models involving a modification in the environment could display changes in gut permeability to some extent. However, to the purpose of investigating mechanisms of disease and suitable treatments, a model should follow at least some of the following criteria (Vervliet and Raes, 2013): (1) face validity, meaning that the modifications observed in animal biochemistry, physiology and/or behavior should resemble those observed in human patients; (2) construct validity, which implies that the etiology of human disease was considered when establishing the model (i.e., alcohol is given to rats to model alcoholic liver disease) and (3) predictive validity, which means that the animal model should respond to interventions (such as pharmacological treatments) in the same way a human patient would. Vervliet and Raes (2013) propose that the relevance of a model relies on its external validity, which according to the authors depends largely on using combined evaluations of predictive, diagnostic and construct validity. In our opinion, depending on the particular use of a model (mechanistic studies, screening of therapeutic compounds, etc.) we could choose two or even one criteria of validity; however reaching all three would provide a more robust model.

With regards to gut barrier alterations, most animal models share a common feature: they display enhanced epithelial permeability. Rodents are typically used, although other animals, such as porcine (Lalles et al., 2007), equine (Marshall and Blikslager, 2011) and avian models (Jeurissen et al., 2002; Baxter et al., 2017), have also been described, to name a few. The last three are particularly useful to investigate veterinary conditions including early weaning, anti-inflammatory drug, feed contamination or restriction, respectively. However there are also reports indicating the suitability of these species to model human diseases that are associated with disrupted gut permeability, as we will mention later.

Intestinal barrier disruption can be achieved by directly intervening on the intestinal environment, or indirectly by targeting another system that communicates to the gut. The latter is the basis for stress- or lesion-induced models that display altered gut permeability. In some cases, however, it is difficult to discern whether a combined effect has been achieved: for example, DSS (an agent that can be added to the animal’s drinking water) is commonly used to chemically damage the mouse or rat intestinal epithelium inducing epithelial leakiness and an inflammatory colitis that resembles IBD in humans (Randhawa et al., 2014), however, it could also indirectly affect the brain-gut axis by causing discomfort and stress to the rodent (Jain et al., 2015), which are known to also enhance intestinal permeability (Meddings and Swain, 2000). Taking this in consideration, the following classification of models refers only to the initial stimuli or intervention applied to the experimental animal (see Table 2 for a further description).

**Table 2:** Examples of experimental approaches to model increased gut permeability.

Species and type of model	Observations	Mechanism proposed by author	Reference
**Intestinal models**			
Mice, oral cadmium exposure (100 mg/L CdCl_2_ for 8 week)	Increased gut permeability and proinflammatory cytokine expression. Decreased tight junction protein expression. Reversed by oral probiotic administration	Genotoxicity, death of epithelial cells, damage to tight junctions, gut dysbiosis	Zhai et al., 2016
Mice, sepsis induced by CLP	Enhanced permeability to FD4 and increased expression of Claudin 2 and JAM-A. Decreased expression of Claudin 5 and Occludin	NA	Yoseph et al., 2016
Rats, allergy induced by oral administration of ovoalbumin	Erosive damage in small intestine. Increased lactulose/mannitol ratio. Altered morphology of tight junctions and decreased expression of tight junction proteins	Inflammation in intestinal tract during allergy induction by OVA	Chen et al., 2014
Mice, colitis induced by oral administration of DSS (3% for up to 7 days)	Loss of ZO-1 and increased permeability to Evan’s Blue dye	Toxic effect on the colonic epithelial cells and crypts leading to changes in the TJ complex and in mucosal permeability prior to the inflammatory infiltrate	Poritz et al., 2007
Rats, colitis induced by anal infusion of TNBS (20 mg)	Decreased expression of ZO-1 and Occludin in colon. Increased levels of endotoxin in serum and colon	Hapten-induced chronic inflammation	Xu et al., 2018
Swine, rectal mucosal lesions induced by TNBS enema (15 mg/ml, 15 min)	Acute increase in paracellular and transcellular permeability. Disrupted localization of ZO-1 in rectal mucosa	Inflammation associated to overexpression of IL-1β, IL-4, IL-10, IFNα and IFNγ	Bregeon et al., 2016
Mice, infection with *Citrobacter rodentium*	Tight junction disruption associated to attachment of the pathogenic bacteria to the cells	Alteration of claudin-3 localization dependent on the intimate attachment of the pathogen to the colonic enterocytes, and independent on inflammation	Guttman et al., 2006
Chickens, infection with several *Eimeria* species	Increased expression of ovotransferrin in the colon. Increased fecal ovotransferrin levels	Release of pro-inflammatory cytokines due to coccidial-induced inflammatory response in the gut	Goossens et al., 2018
Mice, CLP	Increased plasma bacterial load and TNF expression	Excessive bacterial load leading to septic inflammatory response	Parida et al., 2015
Chickens, 24 h feed restriction	Increased permeability to FD4	Increased levels of plasma corticosterone leading to disruption of gut barrier integrity and local inflammation	Baxter et al., 2017
Rats, low protein diet (4% for 20 days)	Decreased mucosal tight junction protein expression and reduced TEER in the colon.	Changes in epithelial cell differentiation	Eyzaguirre-Velasquez et al., 2017
Rats, high fat diet (45% for up to 6 weeks)	Increased flux of HRP flux in correlation with time on the diet	Early and region-specific changes in the gut microbiota which correlate with alterations in gut epithelial function	Hamilton et al., 2015
**Extraintestinal models**			
Mice, pneumonia induced by *Pseudomonas aeruginosa.*	Enhanced permeability to FD4 and increased expression of Claudin 2 and JAM-A. Decreased expression of Claudin 5, ZO-1 and Occludin	NA	Yoseph et al., 2016
Mice, low-dose ionizing radiation (4 Gy)	Redistribution of tight junctions, adherens junctions and actin cytoskeleton in colon mucosa. Increased permeability to FITC-inulin	Ablation of crypt cell proliferation, mitotic catastrophe, and apoptosis leading to gastrointestinal mucositis	Shukla et al., 2016
Mice, burn injury (85°C, 20% body area)	Increased permeability to FD4. Reduced expression of Claudin 4 and 8	Rapid and systemic inflammatory response, including mesenteric vasoconstriction leading to gut hypoxia and cell death	Earley et al., 2015
Rats, brain injury (20 g from a height of 30 cm)	Increased lactulose/mannitol ratio. This is reversed by oral probiotic administration	Intestinal ischemia and hypoxia. Enhanced cell catabolism resulting in depletion of intestinal mucosa main fuel.	Zhang and Jiang, 2015
**Extraintestinal models**			
Rats, hyperthermia (up to 42.5°C, 90 min)	Increased gut permeability to FD4 and marked intestinal epithelial damage	Reduced blood flow to the GI tract resulting in hypoxia, free radical production, ATP depletion, acidosis, and disruption of intestinal epithelial membranes that results in enterocyte necrosis	Lambert et al., 2002
Mice, acute cold exposure (4°C, 30 min)	Increased permeability to L-arabinose. Increased Claudin 2 mRNA expression	Adjustments to the tight junction in order to increase paracellular permeability to nutrient-sized molecules, to meet enhanced digestive/absorptive demand	Price et al., 2013
Rats, acute restraint stress (2 h)	Transient increase in ileal epithelial permeability to alanthanum tracer. Irregular distribution of occludin and ZO-1	Stress-induced contraction of the actin cytoskeleton increasing the distance between adjacent enterocytes	Mazzon et al., 2002
Rats, water avoidance stress (1 h daily for 7 days)	Decreased TEER, increased flux of HRP and altered expression of Claudin 2, JAM-A and ZO-1 in colon	Sub-inflammatory increase in proinflammatory cytokines which mediates the alteration of TJ protein expression	Hattay et al., 2017
Rats, maternal deprivation (one single 4 h event)	Increased gut permeability to FD4 4 and 8 h after deprivation. Increased bacteria translocation to liver and spleen 10 days after deprivation. This is reversed by RU486 (glucocorticoid receptor antagonist)	Increased corticosterone plasma levels is associated to MLCK-dependent cytoskeleton contraction in epithelial cells	Moussaoui et al., 2014
Mice, intensive treadmill exercise (80% of their VO_2_max until exhaustion)	Increased permeability to FD4, electrogenic ion transport and tissue conductance of the small intestine. Also enhanced apoptosis of small intestinal epithelial cells	Gastrointestinal ischemia-associated alterations in epithelial integrity followed by fast repair processes in the duodenum	Gutekunst et al., 2014


*CLP: cecal ligation and puncture. DSS: dextran sulfate sodium. FD4: fluorescein isothiocyanate-dextran 4,000 Da. HRP: horseradish peroxidase. IFN: interferon. IL: interleukin. JAM: junctional adhesion molecule. MLCK: myosin light chain kinase. NA: not available. TEER: transepithelial electrical resistance. TNBS: trinitrobenzene sulfonic acid. ZO: zonula occludens.*

### Models in Which Barrier Dysfunction Is Established on the Gut

Here, the initial intervention is either applied or originated at the GI lumen. Dietary and microbiota changes can protect the intestinal barrier function, which is the case for glutamine (Li et al., 1994), bacteria-derived lactic acid (Ren et al., 2018) and butyrate (Kelly et al., 2015). Other interventions disrupt the gut barrier, as in the case of DSS (Poritz et al., 2007), TNBS (Bregeon et al., 2016; Xu et al., 2018) or heavy metal supplementation (Zhai et al., 2016), low protein (Eyzaguirre-Velasquez et al., 2017) or high fat diet (Hamilton et al., 2015), infection (Guttman et al., 2006; Goossens et al., 2018) or CLP (Parida et al., 2015). These models can be considered to have good construct validity for organic diseases (i.e., those where morphological and/or biochemical features are altered) where barrier function is compromised. For example, DSS or TNBS supplementation in rodents and IL-10 knockout mice are well known models of colitis (Shi et al., 2014; Li et al., 2018; Xu et al., 2018), which is achieved through different mechanisms: DSS induces epithelial injury with exposure of the lamina propria and submucosa to luminal antigens, resulting in inflammation which ultimately alters the gut barrier; TNBS acts as a hapten and also induces inflammation but through Th1-mediated immune responses, which has been shown both in murine (Low et al., 2013) and swine models (Bregeon et al., 2016). IL-10 knockout mice spontaneously develop bowel inflammation, which is associated to colon dysbiosis (Shi et al., 2014). On the other hand, CLP is used to simulate sepsis (Yoseph et al., 2016).

Human gut biochemical features that are commonly associated with perpetuation of intestinal barrier loss (i.e., local proinflammatory cytokines, proteases, neurotransmitters, pathogen-derived products) have been functionally evaluated by using well described gut-derived cell lines such as Caco-2. Here, a cell monolayer is exposed to cleared supernatants obtained from intestinal biopsies, explants or feces. Later, intestinal barrier markers and permeability parameters can be assessed in the cells (Piche et al., 2009). We believe that similar experimental designs could be used to demonstrate that soluble mediators generated at the animal model gut, which are considered to be involved in epithelial barrier disease mechanism, are functionally relevant.

### Models in Which Gut Barrier Dysfunction Is Established Outside the Gut

Lesion induction by skin burn, head trauma, radiation as well as shock induced by hyper- or hypothermia are all considered indirect interventions that increase intestinal permeability. Similar gut alterations are observed when acute or chronic stress is induced by brief water deprivation, motion restraint, overcrowding or forced swim, among others. In all the above cases, the initial disruption is believed to affect systems that are different from the GI, and therefore gut permeability is indirectly enhanced. From the construct validity point of view, these strategies may be more adequate to model barrier function alterations that are subsequent to previous stress or injury, or functional diseases where no morphological and/or biochemical markers are known yet.

Not every increase in gut permeability is associated to disease; for example, exhausting exercise in athletes is followed by marked increases in gut permeability (Lambert, 2009), without known pathological consequences that aren’t attributable to dehydration or heat stress (de Oliveira et al., 2014). Therefore, an effort should be made to prove causality, especially when the injuring stimuli is not directly related to intestinal physiology in an animal model, but gut permeability is proposed as part of the disease mechanism.

## Concluding Remarks

Empiric evidence obtained from animal models, including not only correlation studies but also mechanistic designs allow us to propose (or refuse) particular aspects of barrier function as potential pathways to disease. For example, altered intestinal permeability is described in many chronic diseases; whether it constitutes a risk factor or a target for developing therapies must be established by means of a proper experimental setup.

The complexity of gut barrier function has a lot to do with its connections to other systems. Most of these are not fully understood and therefore, this research is still at its early years. Concomitant alterations in the microbial community, ENS and local immune system should be taken into consideration when interpreting data from animal models, in order to propose significant mechanisms or effective therapeutic strategies for multifactorial diseases. Some future research aims include:

• Implementing experimental approaches that allow for continuous monitoring of these events, taking into consideration that gut barrier function and especially epithelial permeability are dynamic phenomena.
• Investigating the variability of sign/symptom severity that arises from applying single or multiple stimuli, in order to better represent acute or chronic features of disease.
• Including more detailed characterization by reporting several outputs that reflect different elements and consequences of a gut barrier breach, i.e., permeability tests + intestinal epithelial changes + microbial products in the blood + inflammatory changes in the gut or other organs.
• Minimizing confounding factors such as inconsistencies at sampling timing, unwanted stress, and all environmental variables that can disturb barrier function.

## Glossary

### Dysbiosis

Imbalance in the microbial communities that reside a living organism, either by changes in quantity or quality.

### ENS

Intrinsic nervous system of the GI tract. It regulates vital GI functions, including motility, secretion, local immunity, and tissue repair.

### Microbiota/Microbiome

Microbiota is the collection of bacteria, viruses, fungi, and other microorganisms that reside a living organism. Microbiome is the name given to the genes contained in these microbes.

### Tight Junctions

Multiprotein complexes that join two adjacent cells together to form a barrier. The connection involves transmembrane and scaffold proteins, as well as cytoskeleton components.

### Virome

Collection of viruses and associated nucleic acids that reside a living organism.

## Author Contributions

MJ-P drafted the first version and made Table 1. MJ-P and JB supervised the manuscript. MG-G, CD-Z, JE-V, and CG-A made Table 2. All authors revised the manuscript, approved the final version of the manuscript, and evaluated retrieved papers and their reference lists to identify additional relevant articles.

## Conflict of Interest Statement

The authors declare that the research was conducted in the absence of any commercial or financial relationships that could be construed as a potential conflict of interest.

## Abbreviations

CLP: cecal ligation and puncture
DSS: dextran sulfate sodium
ENS: enteric nervous system
FD4: fluorescein isothiocyanate-dextran 4,000 Da
FITC: fluorescein isothiocyanate
GI: gastrointestinal
HRP: horseradish peroxidase
IBD: inflammatory bowel disease
IL: interleukin
JAM: junctional adhesion molecule
PEG: polyethylene glycol
TEER: transepithelial electrical resistance
TNBS: trinitrobenzene sulfonic acid
ZO: zonula occludens
